# XPS characterization of (copper-based) coloured stains formed on limestone surfaces of outdoor Roman monuments

**DOI:** 10.1186/1752-153X-6-S2-S10

**Published:** 2012-05-02

**Authors:** Anna Maria Salvi, Fausto Langerame, Andrea Macchia, Maria Pia Sammartino, Marisa Laurenzi Tabasso

**Affiliations:** 1 University of Basilicata, Chemistry Department, Viale Ateneo Lucano 10, 85100 Potenza, Italy; 2 University of Rome La Sapienza, Chemistry Department, p.le A. Moro 5, 00185 Roma, Italy

## Abstract

Limestone basements holding bronzes or other copper alloys artefacts such as sculptures, decorations and dedicatory inscriptions are frequently met both in modern and ancient monuments. In outdoor conditions, such a combination implies the corrosion products of the copper based alloy, directly exposed to rainwater, will be drained off and migrate through the porous surfaces, forming stains of different colours and intensities, finally causing the limestone structures to deteriorate.

In this work we have analysed samples from two modern limestone monuments in Rome, the Botticino surfaces of the ‘Vittoriano’ (by G.Sacconi, 1885-1911- Piazza Venezia) and the travertine basement of the ‘Statua dello Studente’ (by A.Cataldi, 1920- University city, La Sapienza), and focussed our investigation on the chemical composition of the copper-stained zones using XPS (X-ray Photoelectron Spectroscopy) as a surface-specific technique.

Based on observations reporting on the structure and bonding at the calcite surfaces we have identified copper complexes and mixed calcium/copper carbonates associated with the stains, as well as the chemical state of other elements therein included, and related the compositional changes with differences in chromatic characteristics and sampling locations.

## Background

Outdoor bronzes are subjected to continual corrosion and dissolution processes, as well reported in literature [1-3]: associated with these processes are the coloured stains often observed on stone surfaces of outdoor monuments as the results of leaching from the attached bronze artefacts exposed to rainfall. It is common, in fact, that the corrosion products, dissolved and washed by rain, can reach portions of the nearby stone surfaces. Since all stones are, to various extents, porous, the rainwater laden with corrosion products enters the capillary net and when the stone starts to dry, those products deposited in a sub-surface volume are giving rise to efflorescence and the appearance of stained patches.

The staining phenomena are not only perceived as an aesthetic problem confined to surfaces but also as promoters of a gradual deterioration that over the long term [4] reduces the legibility of the artefacts and deprives those monuments of their intrinsic (historical, religious or political) value.

It is generally accepted that the main constituent of the corrosion products from bronzes consists of Cu(II) salts [5,6], however, the corrosion behaviour depends both on the specific environment and on the alloy characteristics [6,7] and, therefore, the colour of the copper-based stains is certainly influenced by the presence of associated elements contributing to the weathering process.

In order to eliminate or reduce the damage produced by these patches, suitable cleaning procedures are required that would assure the removal of the surface stain without being too aggressive towards the underneath stone components that, in the case of marble and limestone, are mostly calcite and dolomite (calcium carbonate and calcium and magnesium carbonate respectively).

Research work [8-10] carried at the University of Rome is aimed at setting up a cleaning procedure suitable for architectural surfaces and sculptures, based on different operative phases: a) physical-chemical characterization of the patches with combined analytical techniques; b) laboratory test of different chemicals potentially useful for removing the staining products without damaging the stone substrate; c) ‘in situ’ cleaning with procedures selected in phase b).

Each operative phase should be preparatory to the next one however the experiments are performed iteratively and interactively using continuous feedback controls in order, hopefully, to optimize the whole procedure by means of successive approximations.

This paper deals with phase a) contributing with surface analyses to the physical-chemical characterization already in progress with the aid of combined analytical techniques, including also statistical evaluation by PCA (Principal Component Analysis) [10]. An accurate outcome from phase a) is the necessary start for the success of the subsequent phases, clearly taking into account the anticipated results of phases b) and c), as already said.

In particular, two modern limestone monuments in Rome, the ‘Vittoriano’ (by G. Sacconi, 1885-1911, Piazza Venezia) and the basement of the ‘Statua dello Studente’ (by A. Cataldi, 1920, University city, La Sapienza) were studied using XPS (X-ray Photoelectron Spectroscopy) as an analytical mean of investigations.

XPS, the spectroscopic technique most suitable for surface and near- surface analyses of solid compounds, provides elemental, speciation and semi-quantitative analysis at the nanometres scale, as will be explained in the next sections and, more in details, in additional file 1.

In the near future, for the completion of this study, the XPS results as elaborated in this paper will be further rationalized after comparison with results obtained in parallel with Optical Microscopy, X-ray diffraction, Electronic Microscopy combined with Fluorescence analysis (EDXRF) and Micro-Raman spectroscopy [work in progress].

## Methods

**Figure 1:**
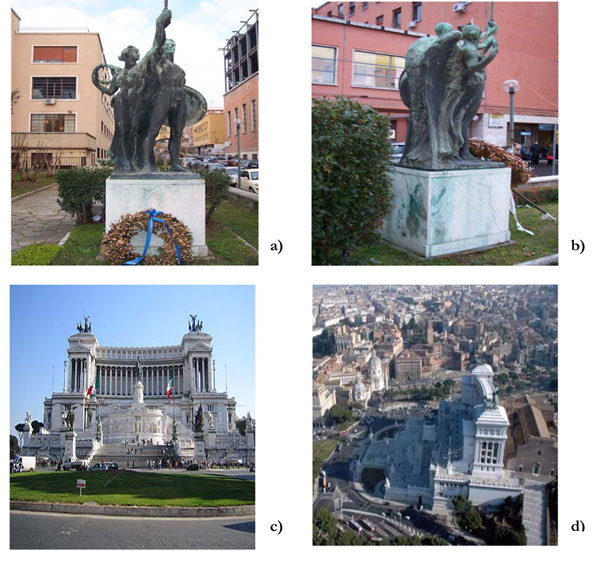
Enlarged views of the two studied monuments in Rome. (a) and (b) ‘Statua dello Studente’ (by A.Cataldi, 1920- University city, La Sapienza); (c) and (d) ‘Vittoriano’ (by G.Sacconi, 1885-1911- Piazza Venezia).

The wide-angle views of the two Roman monuments here investigated, the Student’s Statue (University ‘La Sapienza’) and the ‘Vittoriano’ (Piazza Venezia), are reported in Figures 1a-b and 1c-d, respectively.

Concerning the sampling for XPS analyses, criteria established by the Standards currently in use for the cultural heritage diagnostic were followed: samples of different colours and location were utilized either as small fragments already detached or in form of powders gently ‘scraped’ from the stained surfaces.

The fragments and powders were stored in inert plastic containers, properly plugged (to avoid further contaminants) and tagged with a number indicating the sampling zone: specific details of these zones are given where appropriate in the results section.

The XPS spectra were acquired with a Leybold spectrometer (LH X1) using the achromatic AlKα (1486.6 eV) and MgKα (1253.6 eV) double source at a constant power of 260 W.

Wide and detailed spectra were collected using the FAT (fixed analyzer transmission) mode of operation with a Pass Energy of 50 eV and a channel width of 1.0 and 0.1 eV, respectively. The samples were mounted on the sample holder using a double-sided adhesive copper tape and then transferred to the analysis chamber where the vacuum was always better than 10^-7^ Pa. Care was taken to be sure that no signals from the adhesive tape were visible on the wide spectra.

The wide spectra of each sample were first acquired as a general survey: once the elements composing the sample are identified, the detailed region for each element was then acquired at higher resolution for quantitative (peaks areas) and speciation (chemical states) analysis.

The acquired data were elaborated with a curve-fitting program, NewGoogly [11,12] and the obtained results reported in the tables. The peak assignments (uncertainty on BEs of +/- 0.2 eV) refer to literature data and to the NIST standard reference database available on line (http://srdata.nist.gov/xps/). Peak areas were converted to atomic per cent composition (At%) using established procedures and the appropriate sensitivity factors (SF) [13,14] to assure the correct elemental mass balance, in the limit of our accuracy [13,14]. The energy scales of the XPS figures reported in this paper are not corrected for surface charging but the peak assignments (Binding Energies, BEs), as reported in the tables, are referenced to C1s aliphatic carbon, as an internal standard, set at 285.0 eV. The wide spectra are reported, as acquired, in kinetic energy, whereas the energy scales of the detailed regions are converted to binding energy so as to facilitate comparison of the curve fitted results with literature data.

## Additional materials

A brief description of the basic principles on which XPS is based is provided in Additional File 1 together with information on spectra features and data elaboration.

For the “Vittoriano” samples, supplementary figures, showing C1s, Cu2p3/2, O1s and Ca2p curve-fitted regions, are added as Additional files 2, 3, 4, with the figures caption included.

## Results and discussion

### ‘Statua dello Studente’

**Figure 2:**
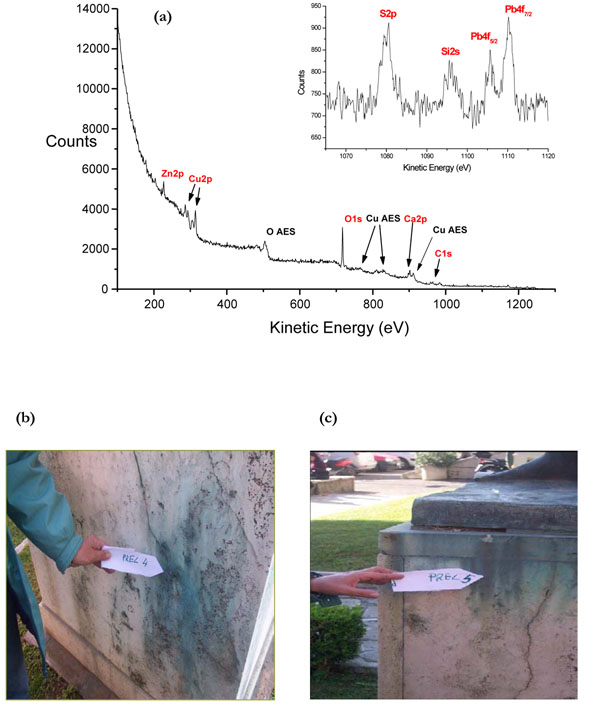
Typical XPS wide spectrum of samples taken from the “Statua dello Studente”(a). Detailed images of the green (b) and grey (c) sampling zones;

The XPS wide spectrum in Figure 2a, acquired with MgKα, is representative of the powdered samples taken from both green and grey zones of the travertine basement where the sampling of the student’s statue was performed, as shown in Figures 2b and c. The labels on the wide spectrum indicate the elements detected and the X-rays induced processes. The red-labelled peaks are directly associated to the photo-emitted electrons (XPS) while the black-labelled peaks (Auger peaks) are due to relaxation processes that follow photoemission always present in XPS spectra (X-AES, X-ray induced Auger Electron Spectroscopy) [13,14]. The utility of both signals, in particular of the Auger parameter α' based on their relative interval in kinetic energy, will be soon evident.

**Figure 3:**
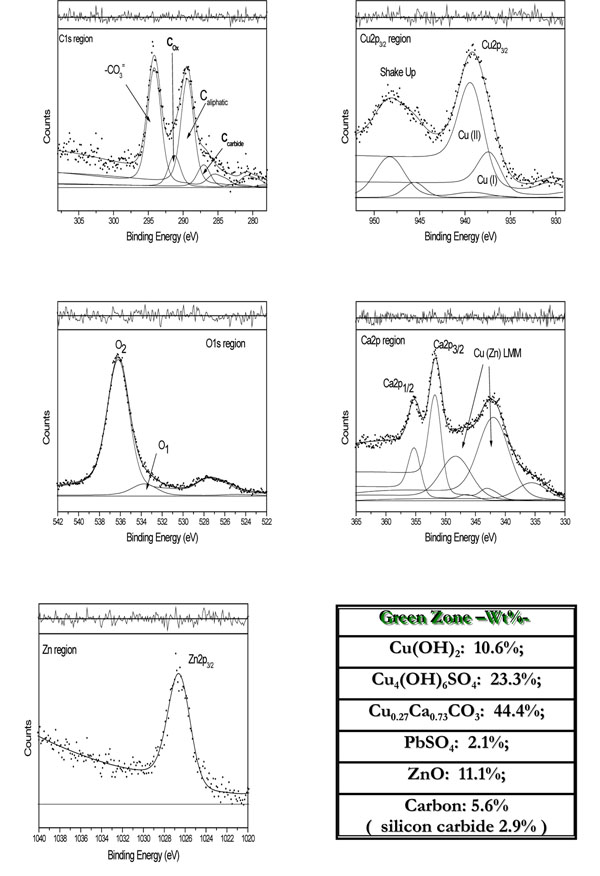
“Statua dello Studente”: C1s, Cu2p3/2, O1s, Ca2p and Zn2p3/2 curve-fitted regions of the green zone and Wt% derived from fitting results reported in Table 1.

**Figure 4:**
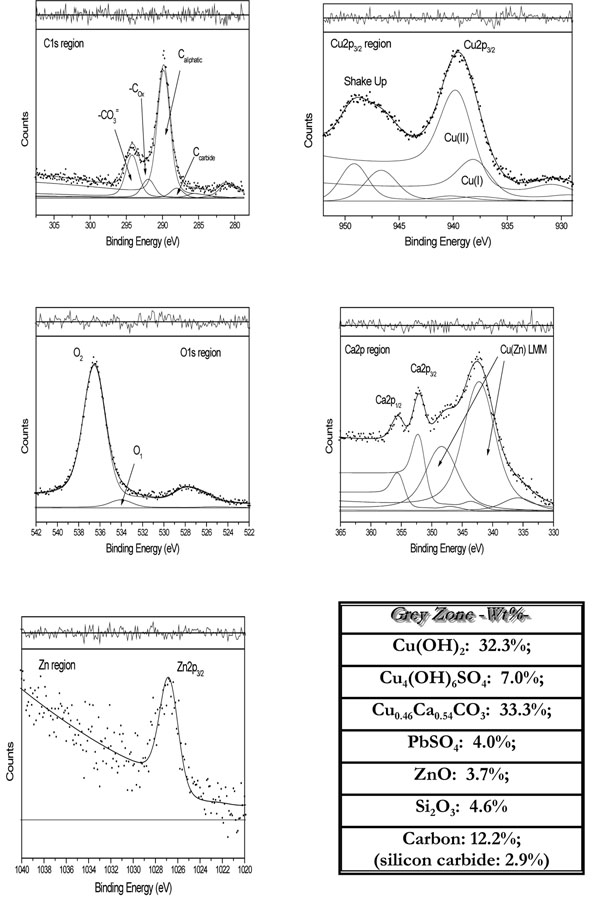
“Statua dello Studente”: C1s, Cu2p3/2, O1s, Ca2p and Zn2p3/2 curve-fitted regions of the grey zone and Wt% derived from fitting results reported in Table 1.

The most significant detailed spectra are respectively shown in Figures 3 - 4 for the two zones together with the weighted percentages, Wt%, of the surface compounds that better represent the results, obtained by curve fitting, summarized in Table 1.

**Table 1:** “Statua dello Studente”: Curve-fitted data of the analyzed regions

	Zone	C_carb_	C-C	C_ox_	CO_3_^2-^	O1s	Ca2p_3/2 #AES_	Cu2p_3/2_	Pb4f_7/2_	S2p	Si2p	Zn2p
**BE_s_**	**GREEN**	282.6	285.0	286.9	289.7	529.4 531.8	347.4^a^	932.9^c^ 934.9	139.0	168.8	102.3	1022.2
**At%**		1.8	8.3	2.4	9.7	54.3	7.1	9.9	0.2	1.4	1.7	3.2
**BE_s_**	**GREY**	282.9	285.0	287.0	289.4	529.6 531.7	347.3^b^	932.8^c^ 934.8	138.8	168.8	102.5	1022.0
**At%**		1.6	20.1	3.0	6.8	47.2	3.6	12.1	0.3	0.6_5_	3.6	1.0_3_

a: # KE (CuLMM) = 915.8 eV α' = 1850.7 eV

b: #KE (CuLMM) = 916.1 eV α' = 1850.9 eV

c: Cu(I): see text

The relative elemental compositions, At%, derived from peak areas are reported for semi-quantitative analysis and Binding Energy, BE, from the peak positions, for qualitative and speciation analysis.

For both zones, the same main elements with similar chemical states were seen to compose the coloured stains. Given the close location of the two zones in the travertine basement this is not surprising, however, differences in the relative abundance of similar compounds, their degree of hydration and the amount of ubiquitous carbonaceous particulates, seem, in concert, responsible for these colour variations and could be taken as indicative of different surface processes taking place at the calcite surface by the action of local corrosion products drained from the statue together with environmental pollutants.

Based on results from curve-fitting, using an XPS online database and cited references, we have made the assignments for each spectral region and cross-checked the peak areas through a combined mass balance i.e. the total area of oxygen from the O1s spectrum to be balanced, in the limit of our accuracy, [13,14] against all the oxygen-containing species, in order to confirm the compounds stoichiometry.

#### C1s

The carbon region, C1s, was curve-fitted with four peaks both for the green and grey zones: see relevant Figures 3-4.

The most prominent peak in the C1s region, C1, at the lower BEs, was assigned to aliphatic carbon set at 285.0 eV and used as an internal standard to correct all binding energies for residual surface charging due to photoemission [13,14].

The C2 peak at around 287 eV was assigned to -C-OH or -C-O species while the C3 peak in the range 289.4-289.7 eV to CO_3_^-2^ in agreement with XPS data on CaCO_3_ polymorphs [15].

The carbon peak at the lowest BE side, seen at 282.6-282.9eV, mostly evident for the green zone, was attributed to carbide species (http://srdata.nist.gov/xps/) given the concomitant presence of Si2p peak at 102.2 eV (Si_2_O_3_/SiC) in these zones.

#### Cu2p

The curve-fitted Cu2p_3/2_ region shows two component peaks plus shake-up satellites. The whole Cu2p region comprises two components, 2p_3/2_ and 2p_1/2_, due to the spin-orbit splitting for orbital having l >1. These two components are well separated (around 20 eV) so only the Cu2p_3/2_ peaks can be considered for the assignment of copper chemical states: the corrected binding energies, derived from Cu2p_3/2_ curve-fitted regions of figures 3-4, are reported in Table 1.

Cu2p_3/2_ (1) at 932.8- 932.9eV was assigned to Cu (I) produced during acquisition (vide infra).

Cu2p_3/2_ (2) at 934.8-934.9eV corresponds, with its shake up satellites, to various Cu (II) species of the type CuSO_4_, CuCO_3_, Cu (OH)_2_. In fact, the quite large peaks width, necessary for curve-fitting, may account for coexistent states very close in energy.

The presence of well evident Auger peaks associated to Cu2p photoemission, has allowed to derive the Auger parameters, α', defined as Cu2p_3/2_ (BE) + Cu LMM (KE) [13,14,16-18].

The Auger parameters, near to 1851, as derived from the peak maxima of both signals reported in Table 1, confirm the above assignments.

As said, the small Cu (I) peak represents the reduction product of Cu (II) under XPS analysis, in conditions generated by the use of achromatic radiations [19] as ours. We have ascertained this fact by repeat experiments, changing the acquisition time (and order) for the Cu2p region. The comparison of spectra, here not reported, has given a confirmation of this phenomenon and of a concomitant readjustment of hydrocarbons in the C1s region. We have taken it into account by acquiring the carbon and copper regions soon after the wide spectra, and followed the same acquisition order, consistently for the all samples.

#### Ca2p

The curve-fitted Ca2p region shows the spin-orbit doublet, Ca2p_3/2_ at 347.3 eV and Ca2p_1/2_ at 350.7eV, having the right intensity ratio (2:1) [13,14] thus indicative of only one chemical state or, as for copper, unresolved closely related chemical states.

In fact, for both zones, the Ca2p_3/2_ BEs at 347.3-347.4eV can be undoubtedly assigned to calcium carbonates [15] but eventual (unresolved) surface oxides, hydroxides or hydrogen carbonates would contribute to the peaks broadening. Given the superimposition of the most intense Cu LMM signals (and of a secondary Zn LMM signal) in the same region some influence on the Ca peaks area can be expected, however, the advantage is that we have an idea of the relative contribution of calcium and copper in the surface depth analysed by XPS and, also, the KE max of the Cu Auger signal is worthy derived by the curve-fitting results (see Table 1).

#### Zn2p

As for copper, the distance (around 24 eV) of the 2p_3/2_ e 2p_1/2_ doublet for zinc is such that only the Zn 2p_3/2_ region was curve-fitted. The corrected BE for Zn2p_3/2_, at ≈1022eV, is typical of ZnO. Literature reports on the likely formation of Zn_5_(CO_3_)(OH)_6_, hydrozincite, following the Zn(II) uptake at calcite surfaces [20], however, from the XPS database the binding energy of Zn2p_3/2_ would be lower, at around 1021.6 eV, in such a case. Thus, ZnO was first confirmed, as preferred assignment, given also the correspondence with the O1 peak at 529.4-529.6eV, O1s region, both regarding the binding energy and the parallel change in intensities. The curve-fitted regions and the results from fitting show this correlation.

#### S2p, Si2s and Pb4f

The three signals of this region were quite noisy, as seen in the inset of Figure 2. The detailed regions are not reported in figures but the results from curve-fitting are reported in Table 1 and hereafter discussed.

The S2p_3/2_ and S2p_1/2_ doublets are very close in energy, around 1eV. Often the S2p region is well fitted with one single peak just for practical reasons therefore, the peak maximum may show a little variability depending on the curve-fitting choice.

In the energy range of 168.8- 169.1eV sulphur can be assigned to SO_4 _^-2^ or to SO_3 _^-2^. Evidence of sulphate reduction to sulphite are reported under XPS analysis [21] but here the signals were of low intensity and thus curve-fitted with only one component and assigned to SO_4 _^-2^.

The corrected position of the Pb4f doublet derived by curve-fitting, Pb4f_7/2_ at 138.8-139.0eV and Pb4f_5/2_ at 143.4-143.6eV, is compatible with PbSO_4_ thus confirming the sulphur assignment, however, in both zones, lead is not sufficient to balance the sulphate and, as reported on the listed weighted compounds (Wt%), the sulphate anions in excess could only be considered as ligands for copper forming mixed complexes with hydroxides.

As for Zn(II), Pb(II) adsorption is reported at calcite surfaces [20]. Thus, (PbCO_3_)_2_(OH)_2_ could be considered as a likely product to fulfil the mass balance with total oxygen, but again, the binding energy of Pb4f_7/2_ would be lower, at around 138eV.

The S2p and Pb4f regions lying close in energy can be acquired together as one single region. This region also contains the Si2s peak that could be curve-fitted to give an estimation of the silicon content in both zones. The areas derived from Si2s and Si2p peaks were the same within the fitting errors, as expected using the proper sensitivity factors for the two spectral regions [13,14].

The Si2p/2s areas were found comparable to the carbide peak, C_carbide_, in the green zone while in the grey zone silicon was found mainly in the oxidized form, Si_2_O_3_: see Table 1.

#### O1s

The O1s region, is fitted with two component peaks at BEs= 529.4-529.6 eV (O1) and BEs = 531.7-531.8 eV (O2), respectively.

The O1 position in the energy scale is characteristic of oxide species. Among the ones here possible, ZnO seems the most probable, given the binding energy of Zn2p_3/2_, however, CaO and CuO could also be eventually considered. We’ll return to this point in the conclusions.

Similarly, the O2 binding energy corresponds to various oxygenated species, as for example, carbonates, sulphates, hydroxides and so on, practically, having similar BEs and thus hardly discernable by curve-fitting. Hence, the usefulness of O1s peaks is on the use of the total area, for balancing all the oxygenated species there contributing, as said above.

On these bases, the compounds listed on Figures 3-4 are those best matching the required mass balance for the green and grey zones, respectively.

As evident, the copper- containing compounds are those prevailing in both coloured zones. Copper (II) ions are present as hydroxyl- and hydroxyl/sulphate-complexes on the limestone outer surface. Most important, in the subsurface, the CO_3 _^-2^ anions are always found in excess with respect to Ca(II) ions, to a different extent for the two zones, thus requiring Cu(II) ions for the net charge balance. As reported from laboratory experiments on the uptake of Cu(II) ions at the calcite surface [22,23], the mixed Ca_x_Cu_1-x_CO_3_ compounds gradually forms following the inclusion of copper ions into the calcite structure. This finding and the related impact on phase c) of the project will be further discussed in the next paragraphs, after having combined the results obtained from both monuments.

### ‘Vittoriano’

The set of samples taken from the Vittoriano surfaces were analyzed with AlKα radiation (1486.6 eV) in order to avoid the superimposition of the Auger signals in the Ca2p region. Spectra were also repeated with the MgKα radiation (1253.6 eV) to verify if the same elements were detected with both sources and for a better comparison with the ‘Student statue’ spectra

**Figure 5:**
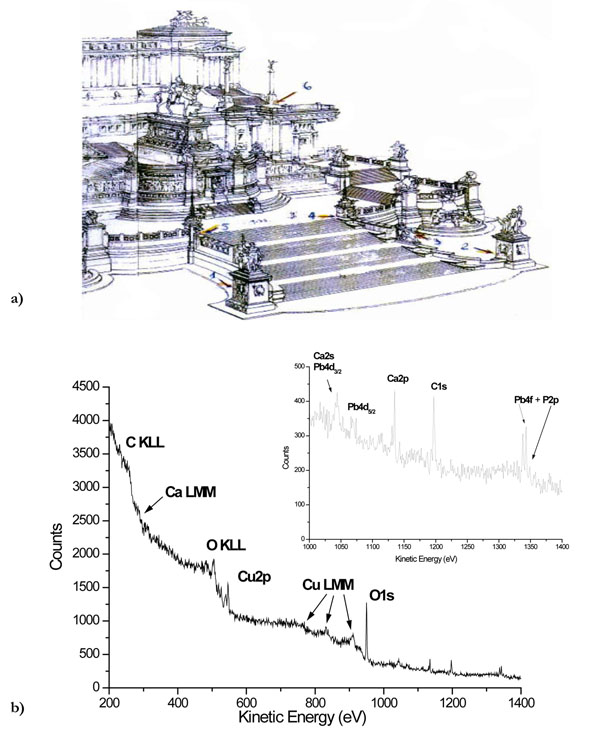
A front view of the ‘Vittoriano Entrance’ (a) [from: *P.L.Porzio* (*editor*), *IL VITTORIANO-Materiali per una storia*, *Soprintendenza per i Beni Ambientali e Architettonici del Lazio*, *Fratelli Palombi Publisher*, *Roma*, *1986]* showing the location of the sampling zones and (b) a typical XPS wide spectrum of the studied Vittoriano’ samples.

In Figure 5a, an enlarged view of the ‘Vittoriano Entrance’ shows the zones where samples were taken for the chemical analysis. In the lower part, Figure 5b, is the wide XPS spectrum representative of the Vittoriano’ samples studied with this surface technique.

**Figure 6:**
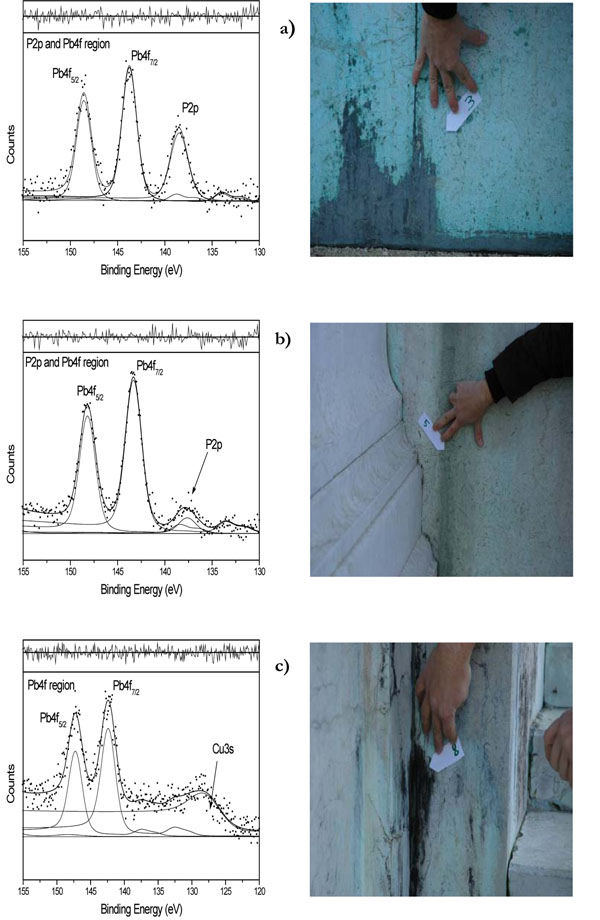
“Vittoriano” sampling points and associated<Pb4f and P2p> curve-fitted regions: **a**) the sampling point (3) zone 2- **b**) the sampling point (5) zone 3- **c)** the sampling point (8) zone 5- see curve- fitting results reported in Table 2.

From just a qualitative point of view, the labelled Ca, Cu, C, O and Pb signals indicate that the main elements are the same as those probed for the ‘Student’ Statue’. The main differences are due to the lack of detection of Zn and S and to the presence of P, Mg and K as new elements, unevenly distributed in the various zones. These differences can be due to the different composition of the two limestones and of the attached bronzes; the relative distance bronze-stone and different urban locations of the two monuments could also play a role. As an example, the correlation of phosphorus with the location of the “Vittoriano” zones is clearly evidenced in Figures 6.

**Table 2:** “Vittoriano”: Curve-fitted data of the analyzed regions

	ZONE	C_carb_	C-C	C_ox_	CO_3_^2-^	O1s	Ca2p_3/2 #AES_	Cu2p_3/2_	K2p	P2p	Pb4f_7/2_	Mg1s
**BE_s_**	**2**	-	285.0	286.4	289.7	531.7 533.9	347.2^a^	933.3^d^/935.6	-	133.9	139.1	1304.5
**At%**		-	10.5	3.1	8.1	58.5	6.5	6.4	-	4.2	0.8	2.2

**BE_s_**	**3**	282.5	285.0	287.4	289.6	531.7	347.1^b^	935.0	-	133.2	139.0	-
**At%**		1.8	29.3	2.0	6.2	42.6	6.5	9.2	-	1.3	1.1	-

**BE_s_**	**5**	281.9	285.0	287.1	289.7	529.5 531.7	347.2^c^	934.3	294.6	-	137.6	-
**At%**		3.0	13.0	3.1	8.8	50.4	5.3	15.0	0.6	-	0.8_5_	-

a: #KE (CuLMM) = 915.4 eV α' = 1851.0 eV

b: #KE (CuLMM) = 915.8 eV α' = 1850.8 eV

c: #KE (CuLMM) = 917.4 eV α' = 1851.7 eV

d: Cu(I): see text

In order to fully understand the similarities and differences, the same detailed investigation for each spectral region was performed (see also supplementary figures A-C and relevant captions) and the curve fitting results hereafter discussed, subdivided for each analysed zone, as reported in Table 2.

#### Zone 2

In Figure 6a are reported the curve-fitted <Pb4f and P2p> regions and the corresponding sampling point of the zone 2, externally located near the pedestrian road.

The supplementary figure A, additional file 2, groups the curve fitted regions of the common elements C, Ca, Cu, O, there detected and shows their chemical states quite similar to those of the previous set as confirmed by results reported in Table 2. Thus, the discussion reported in previous paragraphs, for each spectral region, applies also here, unless otherwise specified.

The samples from zone 2 were light blue-green in colour and found to be the most hydrated both within the Vittoriano set and in comparison to those of the ‘Student’ statue’.

In fact, we see from Table 2 that phosphorus is given at 133.9 eV, a position in binding energies compatible with hydrogen phosphate di-hydrated and, consistently, the O1s region shows a shoulder at around 534eV indicative of hydrating water.

The Mg 1s peak, not reported in figures, is at 1304.5 eV and considered, at first, as a component of the Botticino marble having an intensity ratio of 1:3 with calcium, in agreement with XPS analyses of dolomite surfaces [15]. However, magnesium was found so prominent only in this zone of the Vittoriano, therefore, the possibility of other provenances can not be discarded as will be explained below.

Ca2p and Cu2p_3/2_ peaks have energies still characteristic of carbonates and hydroxides (α’Cu ≈1851), however, the Cu2p_3/2_ is slightly shifted to higher BEs seemingly more influenced by the phosphates anions.

The presence of phosphates, given the location of zone 2, could be ascribed to biological activities: as reported [24], the presence of cornetite, (Cu_3_PO_4_(OH)_4_), was evidenced in studies concerning the effect of bird dropping on outdoor bronzes.

Also lead is found in a chemical state similar to PbSO_4_, in this case, most likely balanced by phosphate anions. The possibility of Pb-complexes with organic ligands should not be disregarded considering the ubiquitous presence of oxygen-containing species in the carbon regions, however, C1s signals in the range of 288-289 eV due to carboxyls, oxalates or amidic groups were not resolved by curve-fitting.

In summary, considering the mass balance with total oxygen, the surface compounds predominating in this sample are mixed carbonates, hydroxides and hydrated phosphates complexes bound with Ca, Cu, Pb and Mg ions, regardless of whether the total amount of magnesium comes only from the dolomite of the underneath stone or if traces from biological residues, released by the surroundings, add to the total amount.

#### Zone 3

In Figure 6b are reported the curve-fitted <Pb4f and P2p> regions and the corresponding sampling point of the zone 3, located at the entrance stairs, above zone 2.

The supplementary figure B, additional file 3, groups the curve fitted regions of the common elements C, Ca, Cu, O detected in this confined area, coloured light grey- with green streaks, as shown in the picture. Looking at the spectra and at the fitting results reported in Table 2, we see that here Mg is not evident or better is not quantifiable- in fact we can see its Auger peak outside the carbon region, but not its photoelectron peak at lower KE. Similarly, we notice that phosphorus is strongly reduced in intensity and at a binding energy closer to that of phosphates with no excess of hydrating water.

These findings may be in support of the hypothesized biological threats around zone 2.

The prominent hydrocarbon components in the carbon region look similar to the grey zone of the student statue, darkening the coloured stains and here obscuring the underlying carbonates even more.

The copper region could here be fitted with one single peak, quite broad to account also for the eventual reduction (Cu(I) peaks not resolved). Consistently with the low content of phosphorus, its binding energy is lowered to 935eV a value also similar to the ‘Student statue’ samples as well as its Auger parameter (α’ = 1850.8eV, Table 2).

The combining of all oxygenated species indicates carbonates, hydroxides and phosphates as complexes for calcium, lead and copper ions. The quite consistent excess of copper (see At%, Table 2) can only be associated to Cu (OH)_2_ for achieving the right mass balance with the total oxygen.

#### Zone 5

In Figure 6c are reported the curve-fitted <Pb4f and P2p> regions and the corresponding sampling point of the zone 5 located internally of the upper stairs, slightly out of reach and better sheltered.

The supplementary figure C, additional file 4, groups the curve fitted regions of the common elements C, Ca, Cu, O composing the black area of this zone 5. The black stains visible in the image are at the junction of two marble slabs, likely a favourite lane for the corrosion products and pollutants, transported by rains, to accumulate.

Here we see that the carbide component in the carbon region is relatively intense but, differently from the student statue samples, silicon is not detected in the surface area or at least is below the detection limit. The binding energy interval of 281.5-283.7 eV accounts for various silicon carbides of SiC_x_ stoichiometry, however, compounds like phenyl- acetylene and cyclic/ unsaturated carbons also are reported to contribute in that region, see NIST database at http://srdata.nist.gov/xps/.

Lead is here at a BE position of PbO/PbO_2_ and phosphorus is below detection, eventually hidden by the background tails of the Cu3s peak. Some traces of oxidized potassium are likely seen, instead. However, the peak at 294.6 eV, not resolved by curve-fitting in the two spin-orbit components, can either be ascribed to K2p for non-stoichiometric oxides or misinterpreted by confusion with energy losses in the C1s background region.

More importantly, the lower Cu2p_3/2_ binding energy at 934.3 eV may include the contribution of tenorite CuO consistently with the satellites peak shape [15] and the change of the Auger parameter now reported at 1851.7 eV (see relevant data in Table 2). Furthermore, the O1s region confirms the presence of oxides either with the O1 peak at 529.5 eV (as it was for ZnO in the green area of ‘Student’ statue) and through the mass balance with the oxygenated species.

Interestingly, the black patches seem to be composed of lead oxide (which, as PbO_2_ Plattnerite, could also contribute to the blackish colour of the patch), mixed calcium and copper carbonate (Ca_0.6_Cu_0.4_CO_3_) with the excess of copper in form of mixed Cu(OH)_2_/CuO in a ratio of 3:1.

## Conclusions

The most important outcomes can be summarized in the following points:

- the major constituent of the coloured stains on both travertine and Botticino limestone is copper – the presence, always in minor quantity, of the other alloys elements in bronze artefacts is dependent on possible differences in their alloy composition and their relative distance and position with respect to the stone surface i.e. zinc was detected by XPS in the basement of the ‘Student statue’ (travertine) but not in the wall surfaces of the ‘Vittoriano’ (Botticino limestone). It should also be noted that tin has never been detected in any of the numerous samples that were analyzed from both the monuments.

- copper is present in form of mixed compounds with calcium: hydroxysulfates, hydroxycarbonates, etc. , the relative amount of counter-anions and different degrees of hydration are related to the location of the monuments in the city and, within the same monuments, to the different sampling zones (fully exposed or partially protected areas)

- the colour of the stains (ranging from light blue-green to grey and dark black) is dependent on the relative amount of the mixed Cu compounds and on the presence of carbonaceous particulates, for example, black patches were found to be composed of tenorite (CuO) normally quite unstable, tending to transform in hydroxycomplexes, but, in this case, probably stabilized in joint zones made impermeable by carbon-containing contaminants. The presence of Plattnerite, PbO_2_, could also significantly contribute to the black colour of these stains.

- Other minor components present in the analysed samples, such as oxidized silicon and amorphous carbon indicate either dissolution of argillaceous inclusions of the carbonate slabs and deposition of air-borne particles [25]: their concomitant presence leads to the formation of silicon oxides with reduced binding energies and carbides, as already detected at the interface of rubber/silica composite materials [26].

The obtained results confirm the tendency of copper leaching from bronzes (transformed by corrosion to Cu(II)) to interact with calcium carbonate and form mixed compound of variable compositions on the surface and sub-surface portions of the limestones. As reported [23], in depth analysis would show the slow formation of a solid solution Ca_x_Cu_1-x_CO_3_, thus very difficult to be removed without affecting the inner stone structure (calcite). Moreover, the presence of CuO in black patches should be considered together with the likely influence of waterproof contaminants [27].

## Competing interests

The authors declare that they have no competing interests.

## Authors' contributions

AMS and FL carried out the XPS measurements, fitted spectra and elaborated data and drafted the manuscript in the light of referenced work. They also provided some basic information on XPS in form of a supplementary appendix.

AM, MPS and MLT conceived the overall research, collected samples and selected the most significant ones to be analyzed by XPS; furthermore, basing on their experience and relevant literature, contributed to the understanding of the experimental results and to the optimization and validation of the manuscript.

All authors have read and approved the final manuscript.

## Supplementary Material

Additional file 1 Appendix Click here for file

Additional file 2 **Figure A** “Vittoriano”: the sampling point (3) of the zone 2’ and relevant C1s, Cu2p3/2, O1s and Ca2p detailed XPS regions - see curve- fitting results reported in Table 2. Click here for file

Additional file 3 **Figure B** “Vittoriano”: the sampling point (5) of the zone 3 and relevant C1s, Cu2p3/2, O1s and Ca2p detailed XPS regions- see curve- fitting results reported in Table 2. Click here for file

Additional file 4 **Figure C** “Vittoriano”: the sampling point (8) of the zone 5 and relevant C1s, Cu2p3/2, O1s and Ca2p detailed XPS regions- see curve- fitting results reported in Table 2. Click here for file

## References

[B1] Chiavari C Rahmouni K Takenouti H Joiret S Vermaut P Robbiola L Composition and electrochemical properties of natural patinas of outdoor bronze monuments Electrochim Acta 2007 52 27 7760 7769 10.1016/j.electacta.2006.12.053

[B2] Serghini-Idrissi M Bernard MC Harrif FZ Joiret S Rahmouni K Srhiri A Takenouti H Vivier V Ziani M Electrochemical and spectroscopic characterization of patinas formed on an archeological bronze coin Electrochim Acta 2005 50 24 4699 4709 10.1016/j.electacta.2005.01.050

[B3] Odnevall I Wallinder Hedberg Y Dromberg P Storm water runoff measurements of copper from a naturally patinated roof and from a parking space. Aspects on environmental fate and chemical speciation Water Res 2009 43 20 5031 5038 10.1016/j.watres.2009.08.025 19762062

[B4] Gaylarde CC Gaylarde PM Beech IB Deterioration of limestone structure associated with copper staining Int Biodeterior Biodegrad 2008 62 2 179 185 10.1016/j.ibiod.2008.01.007

[B5] Ling H Qingrong Z Min G Characterization of corroded bronze Ding from the Yin Ruins of China Corrosion Sci 2007 49 6 2543 2546

[B6] Fitzgerald KP Nairn J Skennerton G Atrens A Atmospheric corrosion of copper and the colour, structure and composition of natural patinas on copper Corrosion Sci 2006 48 9 2480 2509 10.1016/j.corsci.2005.09.011

[B7] Turhan H Adhesive wear resistance of Cu-Sn-Zn-Pb bronze with addition of Fe, Mn and P Mater Lett 2005 59 12 1463 1469 10.1016/j.matlet.2004.11.016

[B8] Laurenzi M Tabasso Macchia A Sammartino MP Visco G SEM/EDS Analysis of Incrustations Coming From the "Fontana delle Tartarughe" (Turtles Fountain) Located in Rome, Italy Chemometrics and Multivariate Analysis Applied to Cultural Heritage and Environment 2006 Nemi (Rome)

[B9] Macchia A Sammartino MP Laurenzi M Tabasso A new method to remove copper corrosion stains from stone surfaces J Archeol Sci 2011 38 6 1300 1307 doi: 10.1016/j.jas.2011.01.005. In press, available online. 10.1016/j.jas.2011.01.005

[B10] Macchia A Salvi AM Sammartino MP Tabasso M Laurenzi Stone and bronze in monuments: decay forms and cleaning proposals Youth in Conservation of Cultural Heritage 2010 Italian Chemical Society, Rome

[B11] Castle JE Salvi AM Chemical state information from the near-peak region of the X-ray photoelectron background J Electron Spectrosc Relat Phenom 2001 114-116 1103 1113

[B12] Castle JE Chapman-Kpodo H Proctor A Salvi AM Curve-fitting in XPS Using Extrinsic and Intrinsic Background Structure J Electron Spectrosc Relat Phenom 1999 106 1 65 80

[B13] Briggs D Seah MP Practical Surface Analysis 1990 1 John Wiley and Sons, Chichester, U.K. Appendix 6 and Chapter 5.

[B14] Briggs D Grant JT Surface Analysis by Auger and X-ray Photoelectron Spectroscopy 2003 IM Publications, Chichester, U.K.

[B15] Gopinath CS Hedge SG Ramaswamy AV Mahapatra S Photoemission studies of polymorphic CaCO3 materials Mater Res Bull 2002 37 7 1323 1332 10.1016/S0025-5408(02)00763-8

[B16] Chawla SK Sankarraman N Payer JH Diagnostic spectra for XPS analysis of Cu-O-S-H compounds J Electron Spectrosc Relat Phenom 1992 16 1 1 18

[B17] Moretti G Auger parameter and Wagner plot in the characterization of chemical states by X-ray photoelectron spectroscopy: A review J Electron Spectrosc Relat Phenom 1998 95 2-3 95 144 10.1016/S0368-2048(98)00249-7

[B18] Malitesta C Sabbatini L Torsi L Zambonin PG Ballivet-Tkatchenko D Galy J Parize JL Savariault JM Copper Speciation by Analytical Electron Spectroscopies: Case of the Intercalation Phase Cu_0.5_V_2_O_5_*0.5H_2_O Surf Interface Anal 1992 19 1-12 513 518 10.1002/sia.740190195

[B19] Iijima Y Niimura N Hiraoka K Prevention of the reduction of CuO during X-ray Photoelectron Spectroscopy Analysis Surf Interface Anal 1996 24 3 193 197 10.1002/(SICI)1096-9918(199603)24:3<193::AID-SIA94>3.0.CO;2-C

[B20] Elzinga EJ Rouff AA Reeder RJ The long-term fate of Cu^2+^, Zn^2+^, and Pb^2+^ adsorption complexes at the calcite surface: An X-ray Absorption spectroscopy study Geochim Cosmochim Acta 2006 70 11 2715 2725 10.1016/j.gca.2006.02.026

[B21] Squarcialupi MC Bernardini GP Faso V Atrei A Rovida G Characterization by XPS of the corrosion patina formed on bronze surfaces J Cult Herit 2002 3 3 199 204 10.1016/S1296-2074(02)01179-2

[B22] Stipp SL Hochella MFJ Structure and bonding environments at the calcite surface as observed with X-ray Photoelectron Spectroscopy (XPS) and low energy electron diffraction (LEED) Geochim Cosmochim Acta 1991 55 6 1723 1736 10.1016/0016-7037(91)90142-R

[B23] Schosseler PM Weahrli B Schweiger A Uptake of Cu^2+^ by the calcium carbonates vaterite and calcite as studied by continuous wave (cw) and pulse elctron paramagnetic resonance Geochim Cosmochim Acta 1999 63 13-14 1955 1967 10.1016/S0016-7037(99)00086-1

[B24] Bernardi E Bowden DJ Brimblecombe P Kenneally H Morselli L The effect of uric acid on outdoor copper and bronze Sci Total Environ 2009 407 7 2383 2389 10.1016/j.scitotenv.2008.12.014 19157513

[B25] Maravelaki-Kalaitzaki P Bertoncello R Biscontin G Evaluation of the initial weathering rate of Istria stone exposed to rain action, in Venice, with X-ray photoelectron spectroscopy Journal of Cultural Heritage 2002 3 273 282 10.1016/S1296-2074(02)01236-0

[B26] Salvi AM Pucciariello R Guascito MR Villani V Intermite L Characterization of the interface in rubber/silica composite materials Surf Interface Anal 2002 33 850 861 10.1002/sia.1463

[B27] Rai US Singh RK Effect of polyacrylamide on the different properties of cement and mortar Mat Sci Eng A-Struct 2005 392 1-2 42 50 10.1016/j.msea.2004.08.050

