# Supplementary material for: XPS characterization of (copper-based) coloured stains formed on limestone surfaces of outdoor Roman monuments
Source: Chem Cent J. 2012 May 2;6(Suppl 2):S10. doi: 10.1186/1752-153X-6-S2-S10 (PMC3342129; doi:10.1186/1752-153X-6-S2-S10)
Supplement: Additional file 1 — Appendix [file 1752-153X-6-S2-S10-S1.doc]

# APPENDIX

# X-ray Photoelectron Spectroscopy

X-ray Photoelectron spectroscopy (XPS) is based on the photoelectric effect introduced by A. Einstein in 1905 [1]. The technique, also known with the acronym ESCA (Electron Spectroscopy for Chemical Analysis), was developed by K. Siegbahn (Uppsala University) who was awarded the Nobel prize in 1981 [2] and further implemented over the years in association with progress in electronic and Ultra High Vacuum (UHV) technologies [3].

Its analytical potential is widely used for the characterization of surfaces. For any materials, generally solids, it provides elemental and chemical state information allowing the qualitative and semi-quantitative composition of their surfaces.

Auger- electron

Photo-electron

*L_2_,L_3_*

*L_1_*

*K*

*1s*

*2s*

*2p*

**Fermi level**

X-rays

**Vacuum level**

*Conduction band*

*Valence band*

**A**

**B**

**Basic principles**

In XPS, the surface of a sample maintained in a ultra high vacuum (10^−10^ mbar) is irradiated with X-rays- in our case- from Mg or Al anodes which provide photons (Kα_1,2_ ) of 1253.6 eV and 1486.6 eV, respectively.

Electrons are ejected from the core levels of atoms in the surface, and their characteristic binding energies **BE**, are determined from the kinetic energy, **KE**, of the ejected electrons and the energy of the incident X-ray beam, **hν** :

**hν = KE + BE**

The kinetic energy of the electrons ejected from the surface is determined by means of an analyzer that, featuring all electrons with a binding energy less than the incident photon energy, gives a photoelectron spectrum. Conducting samples in electrical contact with the sample holder will equilibrate their Fermi level to that of the spectrometer so that the Einstein equation requires to be corrected by the work function, **ϕ,** of the spectrometer and the binding energies to be referred to the Fermi level.

**hν = KE + BE_F_ + ϕ**

If the samples have insufficient conductivity they acquire a surface charge, usually positive, due to the electrons emission under X-ray bombardment and the Einstein equation should be further corrected for this electrostatic charging:

**hν = BE_F_ + KE + (ϕ + S)**

To account for the term in parenthesis and derive the corrected binding energies one of the most reliable methods, for insulating samples, lies on the use of an internal standard whose BE_F_ is known, as for example the C1s of aliphatic carbon set at 285.0eV, in our case (see METHODS).

If the proper charge referencing method is considered, the photoelectron spectrum can give an accurate representation of the electronic structure of an element. The energy shifts due to different chemicals states can be correctly interpreted allowing the assignment of chemical functional groups on the surface.

**Primary and secondary structures**

The characteristic features of an XPS spectrum can be grouped into three basic types: peaks due to photoemission from core levels and valence levels (XPS and VB peaks) and peaks due to X-ray-excited Auger emission (Auger peaks).

When the electron is removed by X-ray irradiation from the core level K contributing to XPS peaks, ***scheme A***, the hole is filled by an electron from L_1_shell, releasing an amount of energy which can appear as X-ray fluorescence or be enough energetic to ionize another electron in the L_2,3_ shell that will then be emitted with a kinetic energy derived from this relaxation process that involves three levels i.e. KLL in ***scheme B.*** This electron is named the Auger electron from its discoverer [4,5] and is easily recognized in the photoelectron spectrum having a kinetic energy independent of the exciting radiation, in contrast to the XPS peaks that obey to the above equations. Thus, if the energy source is changed from MgKα to AlKα, the XPS peaks increase their KE of 233eV while the Auger peaks remain unchanged.

In this work we have focussed our attention to the photoelectron (Cu2p) and Auger (LMM) lines of copper. The shift in energy of the XPS peaks (chemical shifts) indicative of change in chemical states can be even larger for the Auger lines, moreover, their difference in energies was found to be an important source of chemical information being independent of reference level and charging effect [6](see also Auger parameter, *α’*, in the main text and references therein cited)

Associated with primary structures are the so-called secondary structures, often appearing as satellites of the primary peaks. Here we consider the **‘*shake up’* satellites**, important for our work. They arise from the concomitant reorganization of the valence electrons during photoemission of the core electron which may involve their excitation to higher unfilled levels, if available. The primary photoelectrons loose an amount of energy as required for those transitions and, as a result, discrete structures (shake up satellites) appear at the low KE side of the main peak having intensities correlated to the probability of these events.

The shake up satellites are intrinsically related to final states available during photoemission and thus to the atomic number of the elements and their chemical state and structure. For certain transition elements, as for copper in our case, they are dependent on the presence of unpaired electrons in 3d shells. This immediately explains why closed-shell systems (i.e. Cu(I), 3d^10^) have no shake up satellites while open-shell systems (Cu(II), 3d^9^) do.

The strong shake up satellites so evident in our figures have therefore a high diagnostic value and confirm the presence of Cu(II) salts in our samples. Taken together the Cu2p_3/2_ BE values, the Auger parameters (*α’ =* Cu2p_3/2_ (BE) + Cu LMM (KE)) and the relative intensity of the shake up peaks (see figures, text and related comments) helped in discerning the different composition of the copper-based patches.

**Surface-specificity and spectra elaboration**

The wide spectra shown in our paper are composed of a series of peaks sitting on a background that generally increases to low kinetic energy. Electrons have short [mean free path](http://en.wikipedia.org/wiki/Mean_free_path)s in a solid, dependent on their kinetic energies, and the background originates from those having suffered energy losses between photoemission and their detection. With the characteristic X-rays used in conventional XPS, as our double anode, only those electrons created within a depth of 10 nm provide the photoelectron peaks, giving the technique an extreme sensitivity to surface species. The difference in energy between MgKα and AlKα is not so large and, therefore, their sampling depths are not so different either. We have shown that for our samples the same elements were probed with both sources, however, their combined use can be useful for detecting subtle differences at the nanometers scale (not destructive in-depth profiling).

Given the superimposition of background signals, photoelectron and Auger peaks and related satellites, a suitable program for data elaboration should be adopted for deriving the spectral intensity from the peak area measurements.

For samples considered homogeneous within the sampling depth, the spectral intensity of each element is directly proportional to the atomic density and therefore the accuracy in quantification is only as good as the accuracy with which the peak area is measured.

In this work, as for previous XPS work, we used our curve-fitting program, NewGoogly, developed in the course of a longstanding collaboration with the University of Surrey (UK) [7]. The curve-fitting program simultaneously accounts of peaks and related satellites and background parameters. It automatically includes in the fits the contribution of the X-ray satellites due to achromatic sources (see the small peaks, sometimes included in the acquired energy window, located at the high binding energy side of detailed regions, not labelled in the curve-fitted figure).

Once the best fits are obtained, the peaks areas need to be normalized for the sensitivity factors, dependent for each orbital of the given element on both physical (i.e. cross sections, mean free paths) and instrumental (i.e. transmission function) factors. The sensitivity factors used for our spectrometer were experimentally derived, as reported in the references cited in Methods, leading to an overall reproducibility in quantification of the order of 10%.

As reported in Results and Discussion, in the final step of data elaboration, the mass balance is performed using the corrected peak areas in order to determine, in the limit of the given accuracy, the compounds stoichiometry.

**References**

Einstein A. **Über die von der molekularkinetischen Theorie der Wärme geforderte Bewegung von in ruhenden Flüssigkeiten suspendierten Teilchen** (1905) *Annalen der Physik* **17** 549-560

Siegbahn K. **Electron Spectroscopy for Atoms, Molecules and Condensed Matter – an Overview** (1985) *Journal of Electron Spectroscopy & Related Phenomena* **36 (2)** 113-129

Reinert F. & Hüfner S. **Photoemission Spectroscopy – From Early Days to Recent Applications** (2005) *New Journal of Physics* **7** 97

Auger P.(1925) *J.Physic Radium* **6**,205

Lars Persson (1996) *Acta Oncologica* **Pierre Auger-A Life in the Service of Science**. **35** (7): 785–787

Wagner C.D. **Auger Parameter in Electron Spectroscopy for the Identification of Chemical Species** (1975) *Anal.Chem*. **47**, 1201

Salvi A.M. (1996) *PhD Thesis* **The relationship between atomic number and the intensity of the energy loss structure of the photoelectron spectrum** University of Surrey, England
