# Supplementary material for: XPS characterization of (copper-based) coloured stains formed on limestone surfaces of outdoor Roman monuments
Source: Chem Cent J. 2012 May 2;6(Suppl 2):S10. doi: 10.1186/1752-153X-6-S2-S10 (PMC3342129; doi:10.1186/1752-153X-6-S2-S10)
Supplement: Additional file 2 — Figure A “Vittoriano”: the sampling point (3) of the zone 2’ and relevant C1s, Cu2p3/2, O1s and Ca2p detailed XPS regions - see curve- fitting results reported in Table 2. [file 1752-153X-6-S2-S10-S2.doc]

**Figure A** “Vittoriano”: the sampling point (3) of the zone 2’ and relevant C1s, Cu2p3/2, O1s and Ca2p detailed XPS regions - see curve- fitting results reported in Table 2
