# Supplementary material for: XPS characterization of (copper-based) coloured stains formed on limestone surfaces of outdoor Roman monuments
Source: Chem Cent J. 2012 May 2;6(Suppl 2):S10. doi: 10.1186/1752-153X-6-S2-S10 (PMC3342129; doi:10.1186/1752-153X-6-S2-S10)
Supplement: Additional file 4 — Figure C “Vittoriano”: the sampling point (8) of the zone 5 and relevant C1s, Cu2p3/2, O1s and Ca2p detailed XPS regions- see curve- fitting results reported in Table 2. [file 1752-153X-6-S2-S10-S4.doc]

**Figure C** “Vittoriano”: the sampling point (8) of the zone 5 and relevant C1s, Cu2p3/2, O1s and Ca2p detailed XPS regions- see curve- fitting results reported in Table 2
